# Ageism in Higher Education: Historical Reasons and Contemporary Age-Friendly Solutions

**DOI:** 10.1093/geroni/igaa057.1725

**Published:** 2020-12-16

**Authors:** Joann Montepare

**Affiliations:** Lasell University, Newton, Massachusetts, United States

## Abstract

The need for greater attention to aging in higher education is indisputable. Changing age demographics are reshaping societies and challenging colleges and universities to consider how they can respond to aging populations through new approaches to teaching, research, and community engagement. However, ageism permeates academia in systematic as well as implicit ways, holding higher education back from expanding attention to aging. This presentation will describe how ageism manifests itself and how the pioneering Age-Friendly University (AFU) initiative with its ten guiding principles offers a framework to address the neglect of age in academia by advocating for greater age-diversity and inclusion. Special attention will be given to an AFU focus on breaking down age-segregation by bringing younger and older learners together around educational goals of mutual interest, engaging in collaborative teaching and learning experiences, and building intergenerational solidarity in line with social-psychological principles known to reduce prejudice and discrimination. Part of a symposium sponsored by Age-Friendly University (AFU) Interest Group.

